# Risk factors and clinical impact of seroma formation following laparoscopic inguinal hernia repair: a retrospective study

**DOI:** 10.1186/s12893-024-02574-1

**Published:** 2024-10-01

**Authors:** Hong-yang Xie, Bin Chen, Jie Shen, Yi-ping Wang, Wei-cai Shen, Chun-shan Dai

**Affiliations:** Department of Gastrointestinal Surgery, Ningbo Hospital of Traditional Chinese Medicine Affiliated to Zhejiang Chinese Medical University, Ningbo, Zhejiang 315016 China

**Keywords:** Risk factor, Seroma, Laparoscopic, Inguinal hernia

## Abstract

**Background:**

Although laparoscopic inguinal hernia repair (LIHR) has advantages over open surgery, postoperative seroma formation remains an issue. This study aimed to investigate the risk factors and clinical outcomes of seroma formation in patients undergoing LIHR.

**Methods:**

From January 2016 to March 2023, clinical data of patients who underwent LIHR were retrospectively analyzed. Patients who developed seroma and those who did not were classified into the seroma and non-seroma groups, respectively. The demographic and clinical characteristics were compared between the two groups. Univariate and multivariate logistic regression analyses were performed for variables of interest. The receiver operating characteristic curve was used to evaluate the risk factors of the binary logistic model, and the cutoff value for each risk factor was obtained.

**Results:**

Data of 128 patients were evaluated. Compared with patients in the non-seroma group, those in the seroma group had a higher body mass index (BMI) (*P* < 0.001), more direct hernias (*P* < 0.001), larger hernial orifice size (*P* < 0.001), more laparoscopic total extraperitoneal hernioplasty (TEP) (*P* < 0.001), more frequent reduction of hernial sac (*P* = 0.011), and lower preoperative serum albumin level (PSAL) (*P* < 0.001). Multivariate logistic regression analyses performed on these variables showed that high BMI (*P* = 0.005), large hernial orifice (*P* = 0.001), TEP (*P* = 0.033), and low PSAL (*P* = 0.009) were risk factors for seroma formation. Compared with the non-seroma group, the seroma group exhibited a higher numerical rating scale score for postoperative pain (*P* < 0.001), and longer hospital stays (*P* = 0.032).

**Conclusions:**

BMI (> 24.5 kg/m^2^), hernial orifice size (> 2.5 cm), TEP, and PSAL (< 32.5 g/L) were independent risk factors of postoperative seroma formation in patients who underwent LIHR. Although most seromas resolve spontaneously without surgical intervention, seroma formation results in increased patient pain and prolonged hospital stay.

## Introduction

With the advent of laparoscopic surgery, laparoscopic inguinal hernia repair (LIHR) has become the preferred treatment option due to its minimally invasive nature, rapid recovery, decreased post-operative discomfort, and early return to activities [1, 2]. Postoperative seroma is a common complication after LIHR, occurring at a higher rate compared with open techniques [3, 4]. Seroma is the abnormal accumulation of serous fluid in a dead space containing lymphatic and plasma fluids. Although its etiology remains unclear, it is thought to be an accumulation of serum resulting from the disruption of lymphatic and vascular drainage due to extensive tissue dissection during surgery, as well as possible inflammatory exudation, postoperatively collected in a dead cavity [5]. Seroma formation after LHIR manifests as a mass in the hernia region, mimicking hernia recurrence, leading to patient anxiety and adversely affecting the quality of life after surgery [6]. Physical examination alone is insufficient for accurate diagnosis, necessitating the use of ultrasonography or computed tomography (CT) for confirmation [7, 8]. 

Despite the prevalence of seroma following LIHR, studies examining the potential causes and outcomes of this complication is scarce. Therefore, in this study, we aimed to investigate the risk factors and clinical impact of seroma formation in patients undergoing LIHR.

## Methods

### Case selection

Hospital records were retrospectively reviewed to identify patients who underwent LIHR between January 2016 and March 2023. The eligibility criteria included an inguinal hernia diagnosis confirmed via ultrasonography or CT, age ranging from 20 to 80 years, and follow-up record for more than 1 year with complete clinical data. The exclusion criteria included incarcerated or strangulated hernias, blood coagulation disorders, severe hepatic or renal dysfunction, and mental disorders.

The same medical team with expertise in LIHR performed all surgeries. This study was approved by the Ethics Committee of the Ningbo Hospital of Traditional Chinese Medicine Affiliated to Zhejiang Chinese Medical University (No. KYSL-2024-001-027). All procedures were performed in accordance with the tenets of the Declaration of Helsinki. The need for informed consent was waived due to the retrospective nature of the study.

### Surgical procedures

#### Laparoscopic transabdominal preperitoneal hernioplasty (TAPP)

All patients received general anesthesia and were placed in the supine position, tilted approximately 15° to the healthy side. A curved 10 mm incision was made inferior to the umbilicus, and a 10 mm trocar was punctured to establish carbon dioxide (CO_2_) pneumoperitoneum at a pressure of 12 mmHg. A laparoscope was used to investigate the site and size of the inguinal hernia. Two additional 5 mm incisions were made bilaterally at the edges of the abdominal rectus muscles for surgical instruments. An incision was made in the peritoneal wall, starting at the superior margin of the internal inguinal ring. The incision was extended medially to the medial ligament of the umbilicus and laterally to the anterosuperior iliac spine. The peritoneum was dissected away from the abdominal wall to isolate and reduce or transect the hernial sacs. After the spermatic cord in male patients or the round ligament of the uterus in female patients were freed from the peritoneal wall, a knitted polypropylene preformed mesh (BARD 3DMax Light Mesh; Davol, Inc., Warwick, RI, USA) 10.3 × 15.7 cm in size was placed and fixed with tissue adhesive (Borayer, Inc., Nanchang, Jiangxi, China). The peritoneal flap was closed using running absorbable barbed sutures (COVIDIEN, Inc., Mansfield, MA, USA). CO_2_ deflation was performed under observation. Skin incisions were closed using Vicryl sutures (Ethicon, Johnson & Johnson Medical, Cincinnati, OH, USA).

#### Laparoscopic total extraperitoneal hernioplasty (TEP)

All procedures were performed under general anesthesia. A 10 mm infraumbilical incision was made, and the linea alba was identified and incised. A 10 mm trocar was inserted, and a laparoscope was introduced into the preperitoneal space. Blunt separation was performed by placing the tip of the laparoscope toward the pubic symphysis. The CO_2_ pressure was maintained at 12 mmHg. Two 5 mm incisions were made in the midline to introduce the working ports. The preperitoneal working space was established along Cooper’s ligament with blunt dissection medial to the pubic symphysis and lateral to the anterior superior iliac spine. The hernia sac was identified, dissected, and reduced or transected. After the spermatic cord in male patients or the round ligament of the uterus in female patients were freed from the peritoneal wall, a knitted polypropylene preformed mesh (BARD 3DMax Light Mesh, Davol, Inc.) 10.3 × 15.7 cm in size was introduced from the 10 mm port and unrolled to cover the hernia site. CO_2_ deflation was performed under observation. Skin incisions were closed using Vicryl sutures (Ethicon, Johnson & Johnson Medical, Cincinnati, OH, USA).

### Postoperative management

All patients resumed a normal diet and activity a day after surgery. Seroma was defined as fluid collection in the underlying tissue lacunae and in the lacunae formed after surgery due to aseptic inflammatory response and exudate accumulation [3]. The diagnosis of postoperative seroma formation was based on visible swelling of the inguinal canal or scrotum on physical examination and was confirmed via ultrasonography or CT. Surgical intervention for the seroma was considered if patients developed symptoms related to the seroma, such as pain, skin color change, and tenderness.

### Follow-up regimen

The patients were asked to visit the clinic for examination immediately if any discomfort related to hernia repair developed. Regular follow-up was conducted via clinic visits at 1 week, 1 month, 3 months, and 6 months after surgery. Ultrasound was used for follow-up if a seroma developed, and the hospital visit was shortened every 15 days until complete resolution was achieved.

### Data collection

Patients were categorized into the seroma and non-seroma formation groups based on the development of postoperative seroma. The following data were collected: age, sex, smoking, body mass index (BMI), hernia location, type of hernia, scrotal hernia, hernia orifice size, hernia sac size, comorbidity, preoperative medication, recurrent hernia, surgical procedure, operative time, intraoperative bleeding, treatment of the hernia sac, postoperative external compression, and preoperative serum albumin level (PSAL). Postoperative outcomes such as fever, surgical site infection, pain, hospital stay, cost, and recurrence were also documented. The postoperative pain was evaluated using numerical rating scale at 1 h, 12 h, 24 h, and the day of discharge. The mean of the four scores was calculated.

### Statistical analysis

Kolmogorov–Smirnov test was used to test the normality of the continuous data [9]. Data are presented as frequencies or percentages for categorical variables and the mean ± standard deviation for continuous variables. Statistical comparisons of clinical data between the two groups were performed using the Student’s *t*-test for continuous variables and the chi-square test for categorical variables. To investigate the risk factors for seroma formation, we conducted a univariate exact logistic regression analysis for variables that may contribute. Significant variables (*P* < 0.10) were identified for the multivariate analyses. The receiver operating characteristic (ROC) curve was used to evaluate the risk factors of the binary logistic model, and the cutoff value for each risk factor was obtained. Statistical significance was set at values of *P* < 0.05. SPSS (version 17.0; SPSS, Inc., Chicago, IL, USA) was used for statistical analyses.

## Results

**Fig. 1:**
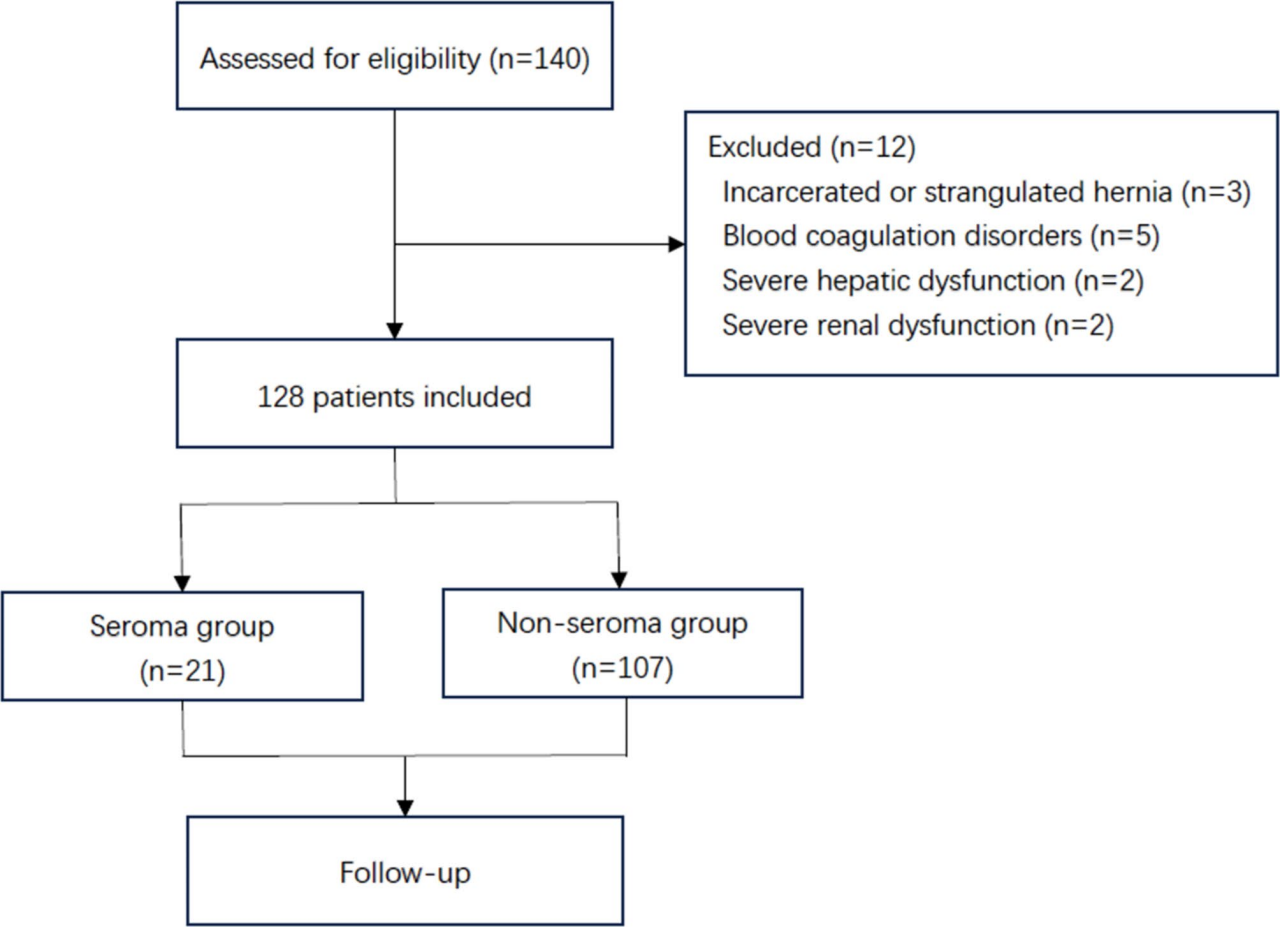
Flowchart of the study

Altogether, 22 female (17.2%) and 106 male (82.8%) patients were included in this study, with an average age of 47.1 ± 12.5 years. There were 21 and 107 patients in the seroma and non-seroma groups, respectively. The study algorithm and analysis process are shown in Fig. 1. No intra-abdominal infections were observed in any patient, and no deaths occurred.

### Risk factors for seroma formation

**Fig. 2:**
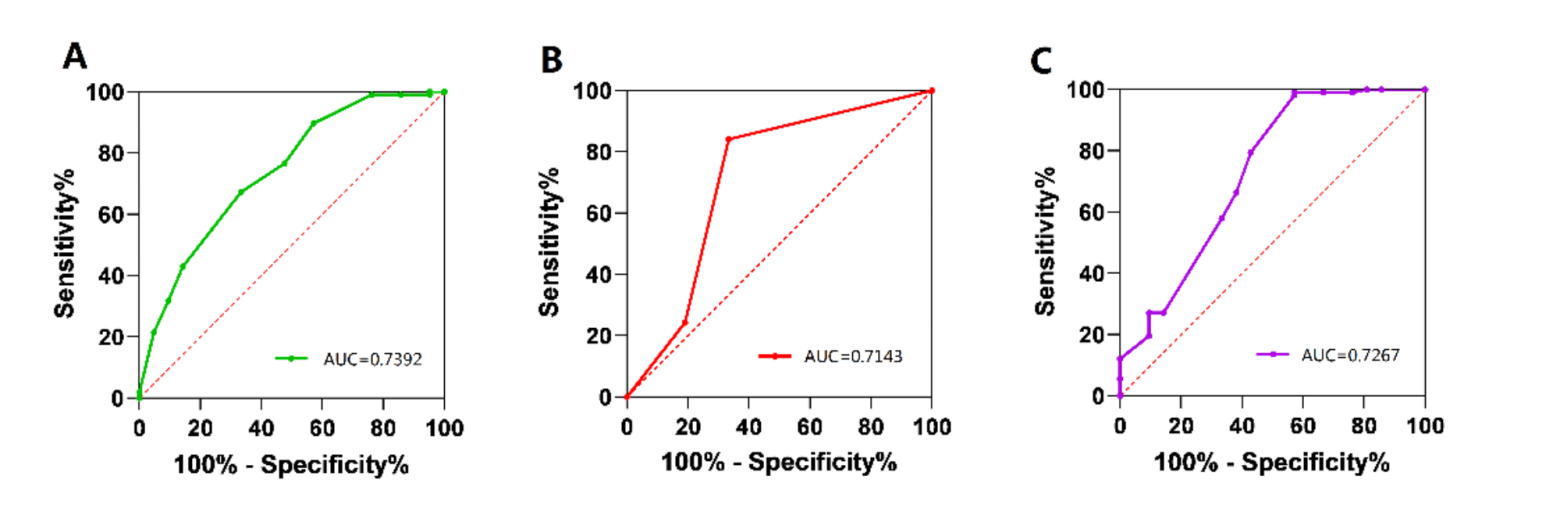
ROC curves of logistic model. ROC = receiver operating characteristic. (**A**, body mass index; **B**, hernial orifice size; **C**, preoperative serum albumin level)

The clinical characteristics and variables of the two groups are summarized and compared in Table 1. No statistically significant differences were found between the seroma and non-seroma groups in terms of age, sex, smoking status, hernia location, scrotal hernia, size of the hernia sac, comorbidities, preoperative medication, recurrent hernia, operative time, intraoperative bleeding, or postoperative external compression (*P* > 0.05). Univariate analysis was conducted on the preoperative variables that demonstrated statistical significance, including BMI, presence of direct hernia, size of hernia orifice, TEP, hernial sac reduction, and PSAL (Table 2). Variables with values of *P* < 0.10 were subjected to further multivariate logistic regression analysis. We found that high BMI > 24.5 kg/m^2^ [odds ratio (OR) 2.557, confidence interval (CI) 2.200-75.599, *P* = 0.005], size of hernia orifice > 2.5 cm (OR 2.653, CI 2.761–72.935, *P* = 0.001), TEP (OR 1.726, CI 1.153–27.371, *P* = 0.033), and PSAL < 32.5 g/L (OR 3.734, CI 2.585-676.894, *P* = 0.009) were confirmed as independent risk factors for seroma formation following LIHR (Table 3). ROC curves were constructed for BMI, hernial orifice size, and PSAL. The areas under the curve were 0.7392, 0.7143, and 0.7267, respectively (Fig. 2).

**Table 1:** Demography and clinical characteristics of patients between the two groups

	Seroma group (*n*= 21)	Non-seroma group (*n*= 107)	*P* value
Age (years)	48.2±16.3	46.9±11.6	0.664
Sex (n, %)			0.805
Male	17 (81.0%)	89 (83.2%)	
Female	4 (19.0%)	18 (16.8%)	
Smoking (n, %)	5 (23.8%)	11 (10.3%)	0.140
BMI (kg/m2)	24.1±2.4	20.9±2.3	<0.001
Hernia location			0.346
Left side	6 (28.6%)	24 (22.4%)	
Right side	12 (57.1%)	76 (71.0%)	
Bilateral sides	3 (14.3%)	7 (6.5%)	
Type of hernia (n, %)			<0.001
Direct hernia	10 (47.6%)	12 (11.2%)	
Indirect hernia	11 (52.4%)	95 (88.8%)	
Scrotal hernia (n, %)	4 (19.0%)	9 (8.4%)	0.226
Hernial orifice size (cm)	2.8±0.7	1.8±0.6	<0.001
Hernial sac size (cm)	3.0±0.8	3.0±0.8	0.986
Comorbidity (n, %)			0.263
Hypertension	4 (19.0%)	20 (18.7%)	
COPD	1 (4.8%)	12 (11.2%)	
Renal insufficiency	5 (23.8%)	12 (11.2%)	
Diabetes	6 (28.6%)	19 (17.8%)	
None	5 (23.8%)	44 (41.1%)	
Preoperative medication			0.106
Antiplatelet therapy	4 (19.0%)	9 (8.4%)	
Anticoagulation therapy	6 (28.6%)	18 (16.8%)	
None	11 (52.4%)	85 (79.4%)	
Recurrent hernia (n, %)	2 (9.5%)	9 (8.4%)	1.000
Surgical procedure			<0.001
TEP (n, %)	15 (71.4%)	12 (11.2%)	
TAPP (n, %)	6 (28.6%)	95 (88.8%)	
Operative time (min)	68.6±10.3	65.4±11.9	0.253
Intraoperative bleeding (mL)	10.2±6.7	11.9±4.8	0.195
Hernial sac treatment			0.011
Reduction	9 (42.9%)	19 (17.8%)	
Transection	12 (57.1%)	88 (82.2%)	
Postoperative external compression	3 (14.3%)	11 (10.3%)	0.712
PSAL (g/L)	31.9±6.7	39.6±6.2	<0.001

BMI, body mass index; COPD, chronic obstructive pulmonary disease; TAPP, laparoscopic transabdominal preperitoneal hernioplasty; TEP, laparoscopic total extraperitoneal hernioplasty; PSAL, preoperative serum albumin level

**Table 2:** Univariate logistic regression for risk factors for seroma formation following laparoscopic inguinal hernia repair

	B	S.E.	Wals	OR	95%CI	*P* value
BMI (> 24.5 kg/m^2^)	1.879	0.544	11.934	6.545	2.254–19.005	<0.001
Direct hernia	1.974	0.534	13.679	7.197	2.529–20.482	<0.001
Hernial orifice size (> 2.5 cm)	2.360	0.533	19.592	10.588	3.724–30.104	0.001
TEP	2.985	0.572	27.237	19.792	6.450-60.726	<0.001
Hernial sac reduction	1.245	0.508	6.000	3.474	1.283–9.408	0.014
PSAL (< 32.5 g/L)	3.673	0.839	19.165	39.375	7.604-203.893	<0.001

BMI, body mass index; PSAL, preoperative serum albumin level; TEP, laparoscopic total extraperitoneal hernioplasty

**Table 3:** Multivariate logistic regression for risk factors for seroma formation following laparoscopic inguinal hernia repair

	B	S.E.	Wals	OR	95%CI	*P* value
BMI (> 24.5 kg/m^2^)	2.557	0.902	8.031	12.897	2.200-75.599	0.005
Direct hernia	-0.763	1.195	0.408	0.466	0.045–4.848	0.523
Hernial orifice size (> 2.5 cm)	2.653	0.835	10.088	14.191	2.761–72.935	0.001
TEP	1.726	0.808	4.561	5.617	1.153–27.371	0.033
Hernial sac reduction	1.318	0.939	1.972	3.736	0.593–23.523	0.160
PSAL (< 32.5 g/L)	3.734	1.420	6.910	41.834	2.585-676.894	0.009

BMI, body mass index; PSAL, preoperative serum albumin level; TEP, laparoscopic total extraperitoneal hernioplasty

### Outcomes of seroma formation after LIHR

Both groups exhibited no significant differences in postoperative fever, wound infection, hospitalization costs, and recurrence (*P* > 0.05). However, the seroma group had a higher numerical rating scale of postoperative pain (2.2 ± 1.1 vs. 1.5 ± 0.6, *P* < 0.001) and a longer hospital stay (3.5 ± 1.4 vs. 3.0 ± 0.9, *P* = 0.032) than those of the non-seroma group (Table 4). In the seroma group, all patients were diagnosed within 1 week postoperatively, and seroma formation occurred 4.1 ± 1.3 days after LIHR. Four patients underwent surgical drainage due to pain, tenderness, and increased seroma size at 8.2 ± 1.5 days postoperatively. The resolution of seromas was complete at 81.0 ± 10.7 days and the resolution rate of the cohort within 3 months was 95.2%.

**Table 4:** Comparison of outcomes between the two groups

	Seroma group (*n* = 21)	Non-seroma group (*n* = 107 )	*P* value
Postoperative fever (n, %)	2 (9.5%)	6 (5.6%)	0.498
NRS of postoperative pain	2.2 ± 1.1	1.5 ± 0.6	<0.001
Wound infection (n, %)	1 (4.8%)	2 (1.9%)	0.423
Hospital stay (days)	3.5 ± 1.4	3.0 ± 0.9	0.032
Hospitalization cost (dollars)	1472.9 ± 183.9	1481.7 ± 117.7	0.779
Recurrence (n, %)	1 (4.8%)	0	0.164

NRS, numerical rating scale

## Discussion

Compared with open approaches, LIHR offers advantages, including lesser postoperative pain, lesser consumption of analgesics, shorter recovery time, and earlier return to work [10, 11]. However, it is associated with higher incidence of seroma formation in LIHR, which remains challenge for surgeons [12]. 

Some studies have demonstrated that a high BMI is associated with postoperative complications and a prolonged length of hospital stay [13–16]. However, the relationship between high BMI and seroma formation after LIHR remained unclear. This study revealed that BMI > 24.5 kg/m^2^ is a risk factor for seroma. A raised BMI is associated with challenges in surgical care that occur both directly and indirectly because of the elevated BMI. These can result in significant problems in the surgical procedure including inguinal hernia repair [17]. In our experience, patients with a high BMI usually have more fat tissue in the inguinal region results in additional effort required to strip the hernia sac, thereby increasing postoperative wound exudation.

In our study, a hernial orifice > 2.5 cm was considered a risk factor for postoperative seroma formation, consistent with Morito et al.’s study [18]. Generally, large hernial orifices are associated with severe tissue adhesion and thickening. In these cases, dissection of hernial sac is difficult and prone to accidental damage to the spermatic cord blood vessels. Therefore, a large hernial defect tends to complicate and prolong LIHR, causing increased intraoperative bleeding and postoperative wound exudation. Furthermore, a large postoperative cavity is created after hernia is reduced. All these aforementioned factors contribute to the development of postoperative seromas.

Our findings revealed a higher incidence of seromas in the TEP group than in the TAPP group. This disparity can be attributed to several factors. TEP separation requires creating a preperitoneal working space from the umbilicus to the pubic symphysis, which is more extensive than that in TAPP. TEP not only causes greater trauma and increased postoperative tissue fluid exudation but also creates a dead space through the dissection and reduction of the hernial sac after surgery. Furthermore, the closed cavity is the lowest part of the inguinal region and is prone to fluid accumulation, and drainage of exudates into other natural body cavities is impossible. In contrast, small gaps in the peritoneum after peritoneal closure in TAPP enable the drainage of preperitoneal exudation into the peritoneal cavity, remarkably decreasing the possibility of seroma formation.

In surgery, it is well established that a low PSAL is associated with a greater risk of adverse surgical outcomes such as infection, edema, and wound dehiscence [19–21]. PSAL is predictive of operative outcomes because it is a marker of disease and malnutrition and confers a direct protective effect through biological mechanisms [22, 23]. This study showed that patients with low PSAL had a higher occurrence of seroma formation following LIHR. A low PSAL causes a decrease in colloid osmotic pressure, which promotes fluid transfer from the intravascular to the extravascular and tissue interstitial spaces. This etiology worsens the edema of the damaged postoperative tissue, resulting in increased exudation and seroma formation.

In this study, we identified several risk factors for seroma formation following LIHR. However, the exact mechanism underlying seroma formation remains poorly understood, with several hypotheses. Some studies have demonstrated that the remaining large hernial defect space after surgery contributes to the formation of a seroma [24, 25]. In addition, an inflammatory response from surgical dissection and the presence of a polypropylene mesh have been reported to cause seromas [26]. Accordingly, various strategies have been adopted to reduce the incidence of postoperative seromas. These include drainage tubes, intraoperative hypertonic saline irrigation, and pressure dressings [4, 27–30]. Postoperative external compression facilitates dead space obliteration, avoids surgical site shearing, and promotes tissue adhesion. However, pressure bandages are difficult to apply over the groin, and it did not reduce the incidence of postoperative seromas in our study.

Seroma formation results in patient dissatisfaction and poor aesthetic outcomes [31]. Moreover, our study demonstrated that it causes increased postoperative pain and prolongs hospital stays. Expectant treatment with observation is plausible in small seromas, which are mostly absorbed within 3 months. However, several symptomatic seromas require postoperative surgical drainage.

This study has some limitations. First, it has a retrospective and single-center design. Second, the patient population is relatively small. Third, selection bias existed because not all patients routinely underwent postoperative CT or ultrasound and some asymptomatic patients with seromas may have been ignored. Therefore, a randomized, controlled, and multicenter study with a large sample size is required for further investigation.

## Conclusions

The independent risk factors for postoperative seroma formation in patients who underwent LIHR were BMI (> 24.5 kg/m^2^), hernial orifice size (> 2.5 cm), TEP, and PSAL (< 32.5 g/L). Most seromas resolve spontaneously; however, seroma formation results in increased pain and prolonged hospital stay.

## Data Availability

The datasets generated and/or analyzed during the current study are not publicly available due to the protection of personal privacy but are available from the corresponding author on reasonable request.

## Abbreviations

LIHR: Laparoscopic inguinal hernia repair
BMI: Body mass index
TEP: Laparoscopic total extraperitoneal hernioplasty
PSAL: Preoperative serum albumin level
TAPP: Laparoscopic transabdominal preperitoneal hernioplasty
CT: Computed tomography

## References

[1] Shah MY, Raut P, Wilkinson TRV, Agrawal V. Surgical outcomes of laparoscopic total extraperitoneal (TEP) inguinal hernia repair compared with Lichtenstein tension-free open mesh inguinal hernia repair: a prospective randomized study. Med (Baltim). 2022;101(26):e29746. 10.1097/MD.0000000000029746 PMC9239617 35777031

[2] Pulikkal Reghunandanan R, Ali Usman A, Basheer S, Kuttichi L, Els Jojo J. Abdul Rasheed MF. Laparoscopic Versus Open Inguinal Hernia Repair: a comparative study. Cureus. 2023;15(11):e48619. 38090402 10.7759/cureus.48619 PMC10711334

[3] Pan C, Xu X, Si X, Yu J. Effect of complete reduction of hernia sac and transection of hernia sac during laparoscopic indirect inguinal hernia repair on seroma. BMC Surg. 2022;22(1):149. 35468781 10.1186/s12893-022-01599-8 PMC9036776

[4] Fang H, Lin R, Lin X, Lu F, Yang Y, Wang C, et al. Drainage decreases the seroma incidence in laparoscopic transabdominal preperitoneal (TAPP) hernia repair for large inguinoscrotal hernias. Asian J Surg. 2021;44(3):544–8. 33191072 10.1016/j.asjsur.2020.11.003

[5] Kazzam ME, Ng P. Postoperative Seroma Management. StatPearls. Treasure Island (FL) ineligible companies. Disclosure: Paul Ng declares no relevant financial relationships with ineligible companies.: StatPearls Publishing Copyright © 2024. StatPearls Publishing LLC.; 2024. 36256748

[6] Lyu Y, Cheng Y, Wang B, Du W, Xu Y. Comparison of endoscopic surgery and Lichtenstein repair for treatment of inguinal hernias: a network meta-analysis. Med (Baltim). 2020;99(6):e19134. 10.1097/MD.0000000000019134 PMC7015567 32028439

[7] Robinson A, Light D, Kasim A, Nice C. A systematic review and meta-analysis of the role of radiology in the diagnosis of occult inguinal hernia. Surg Endosc. 2013;27(1):11–8. 22733195 10.1007/s00464-012-2412-3

[8] Kamei N, Otsubo T, Koizumi S, Morimoto T, Nakajima Y. Prone computed tomography hernia study for the diagnosis of inguinal hernia. Surg Today. 2019;49(11):936–41. 31243553 10.1007/s00595-019-01837-2

[9] Mishra P, Pandey CM, Singh U, Gupta A, Sahu C, Keshri A. Descriptive statistics and normality tests for statistical data. Ann Card Anaesth. 2019;22(1):67–72. 30648682 10.4103/aca.ACA_157_18 PMC6350423

[10] Haladu N, Alabi A, Brazzelli M, Imamura M, Ahmed I, Ramsay G, et al. Open versus laparoscopic repair of inguinal hernia: an overview of systematic reviews of randomised controlled trials. Surg Endosc. 2022;36(7):4685–700. 35286471 10.1007/s00464-022-09161-6 PMC9160137

[11] Tadaki C, Lomelin D, Simorov A, Jones R, Humphreys M, daSilva M, et al. Perioperative outcomes and costs of laparoscopic versus open inguinal hernia repair. Hernia. 2016;20(3):399–404. 26874507 10.1007/s10029-016-1465-y

[12] Reddy VM, Sutton CD, Bloxham L, Garcea G, Ubhi SS, Robertson GS. Laparoscopic repair of direct inguinal hernia: a new technique that reduces the development of postoperative seroma. Hernia. 2007;11(5):393–6. 17541495 10.1007/s10029-007-0233-4

[13] van Vugt JL, Cakir H, Kornmann VN, Doodeman HJ, Stoot JH, Boerma D, et al. The new body Mass Index as a predictor of postoperative complications in elective colorectal cancer surgery. Clin Nutr. 2015;34(4):700–4. 25171837 10.1016/j.clnu.2014.08.006

[14] Wang H, Jin J, Zhu F, Peng F, Wang M, Qin R. The ratio of abdominal depth to body mass index is a preoperative predictor of postoperative complications after laparoscopic pancreaticoduodenectomy: a retrospective propensity score matched analysis. Surg Endosc. 2021;35(12):6472–80. 33156385 10.1007/s00464-020-08140-z

[15] Sadok N, Krabbe-Timmerman IS, de Bock GH, Werker PMN, Jansen L. The Effect of Smoking and Body Mass Index on the complication rate of alloplastic breast Reconstruction. Scand J Surg. 2020;109(2):143–50. 30712467 10.1177/1457496919826711

[16] McLoughlin LC, Kassouf W, Breau RH, Fairey A, Agnihotram VR, Salimi A, et al. Obesity and complication risk from Radical Cystectomy: identifying a body Mass Index threshold. J Urol. 2023;209(1):111–20. 36250946 10.1097/JU.0000000000002988

[17] Olugbemi M, Athisayaraj T, Lorejo E, Coveney E. The impact of body Mass Index on local anaesthetic inguinal hernia repair. Cureus. 2023;15(3):e36163. 37065380 10.7759/cureus.36163 PMC10102715

[18] Morito A, Kosumi K, Kubota T, Yumoto S, Matsumoto T, Mima K, et al. Investigation of risk factors for postoperative seroma/hematoma after TAPP. Surg Endosc. 2022;36(7):4741–7. 34713342 10.1007/s00464-021-08814-2

[19] Naga Rohith V, Arya SV, Rani A, Chejara RK, Sharma A, Arora JK, et al. Preoperative Serum Albumin Level as a predictor of Abdominal Wound-related complications after emergency exploratory laparotomy. Cureus. 2022;14(11):e31980. 36589182 10.7759/cureus.31980 PMC9797030

[20] Seretis C, Gill J, Malik A, Elhassan AM, Shariff U, Youssef H. Low preoperative serum albumin levels are Associated with impaired outcome after cytoreductive surgery and Perioperative Intraperitoneal Chemotherapy for Peritoneal Surface malignancies. J Clin Med Res. 2020;12(12):773–9. 33447310 10.14740/jocmr4362 PMC7781284

[21] Rong G, Liu S, Xi C, Wang C, Deng J, Qin T. Correlation between preoperative serum albumin-globulin ratio and prognosis of patients undergoing low rectal Cancer surgery. Clin Lab. 2022;68(1). 10.7754/Clin.Lab.2021.210213 35023685

[22] Heise D, Bednarsch J, Kroh A, Schipper S, Eickhoff R, Lang S, et al. Operative time, Age, and serum albumin Predict Surgical Morbidity after laparoscopic liver surgery. Surg Innov. 2021;28(6):714–22. 33568020 10.1177/1553350621991223 PMC8649428

[23] Hart A, Sun Y, Titcomb TJ, Liu B, Smith JK, Correia MLG, et al. Association between preoperative serum albumin levels with risk of death and postoperative complications after bariatric surgery: a retrospective cohort study. Surg Obes Relat Dis. 2022;18(7):928–34. 35660268 10.1016/j.soard.2022.04.006 PMC11406824

[24] Lau H, Lee F. Seroma following endoscopic extraperitoneal inguinal hernioplasty. Surg Endosc. 2003;17(11):1773–7. 12802655 10.1007/s00464-002-8771-4

[25] Matsumoto R, Nagahisa Y, Hashida K, Yokota M, Okabe M, Kawamoto K. Strangulated Hernia can be a risk factor of Seroma following laparoscopic Transabdominal Preperitoneal Repair. Minim Invasive Surg. 2018;2018:6528075. 30225141 10.1155/2018/6528075 PMC6129322

[26] Bittner R, Schmedt CG, Leibl BJ, Schwarz J. Early postoperative and one year results of a randomized controlled trial comparing the impact of extralight titanized polypropylene mesh and traditional heavyweight polypropylene mesh on pain and seroma production in laparoscopic hernia repair (TAPP). World J Surg. 2011;35(8):1791–7. 21607821 10.1007/s00268-011-1148-x

[27] Dudai M, Gilboa Ittah K. Intraoperative hypertonic saline irrigation preventing seroma formation and reducing drain secretion in extended endoscopic hernia and linea Alba reconstruction glue. Hernia. 2019;23(6):1291–6. 31055707 10.1007/s10029-019-01956-2

[28] Ismail M, Garg M, Rajagopal M, Garg P. Impact of closed-suction drain in preperitoneal space on the incidence of seroma formation after laparoscopic total extraperitoneal inguinal hernia repair. Surg Laparosc Endosc Percutan Tech. 2009;19(3):263–6. 19542859 10.1097/SLE.0b013e3181a4d0e1

[29] Fabro EAN, Teodózio CGC, Costa RM, Macedo FO, Cardoso A, Jacob RBE, et al. Clinical experience with Compression Taping to treat Seroma after breast Cancer surgery: A Medical device clinical study. Adv Skin Wound Care. 2022;35(7):1–6. 35723961 10.1097/01.ASW.0000831068.34587.3d

[30] Hagbevor I, Ali MA, Awuku GA. Closed non-suction drain placement as haematoma and seroma formation preventive measure post-nylon darn surgery for inguinoscrotal hernias in adults. Hernia. 2022;26(1):123–30. 34115244 10.1007/s10029-021-02430-8 PMC8881237

[31] Petersen M, Friis-Andersen H, Zinther N. Does closure of the direct hernia defect in laparoscopic inguinal herniotomy reduce the risk of recurrence and seroma formation? A systematic review and meta-analysis. Hernia. 2023;27(2):259–64. 36495351 10.1007/s10029-022-02724-5

